# Improving the quality of cause of death data for public health policy: are all ‘garbage’ codes equally problematic?

**DOI:** 10.1186/s12916-020-01525-w

**Published:** 2020-03-09

**Authors:** Mohsen Naghavi, Nicola Richards, Hafiz Chowdhury, James Eynstone-Hinkins, Elisabeth Franca, Michael Hegnauer, Ardeshir Khosravi, Lauren Moran, Lene Mikkelsen, Alan D. Lopez

**Affiliations:** 1 Institute of Health Metrics and Evaluation, University of Washington, 2301 5th Ave, Seattle, WA 98121 USA; 2 Melbourne School of Population and Global Health, University of Melbourne, 207 Bouverie Street, Carlton, VIC 3053 Australia; 3 Australian Bureau of Statistics, 295 Ann Street, Brisbane, QLD 4000 Australia; 4 University of Minas Gerais, Belo Horizonte, Minas Gerias 31270-901 Brazil; 5 Swiss Tropical and Public Health Institute, University of Basel, Basel, Switzerland; 6 Ministry of Health and Medical Education, District 2, Eyvanak Blvd, Tehran, Iran

**Keywords:** Mortality, Cause of death, Garbage codes, Unusable and insufficiently specified codes, ICD-10, Ill-defined codes, Policy, Planning

## Background

All countries need accurate and timely mortality statistics to inform health and social policy debates and to monitor progress towards national and global health development goals. In many countries, however, civil registration and vital statistics (CRVS) systems are poorly developed. Consequently, the statistics they produce are not fit for purpose. In part, this arises because the physicians certifying cause of death (COD) have either not been adequately trained in how to complete a death certificate according to the current International Statistical Classification of Diseases – Version 10 (ICD-10) [1], or they fail to appreciate the public health importance of what is often perceived as a largely administrative task [2]. This can be reinforced by cultural attitudes and perceptions among hospital administrators, who are generally unaware of the critical contribution that accurate medical certification of CODs makes to generating essential public health intelligence that can be used for planning.

Unsurprisingly, these system deficiencies usually result in a high proportion of CODs being assigned to ‘garbage’ codes [3]. These have little or no public health value because they are too vague, are an immediate or intermediate COD, or are impossible as an underlying cause of death (UCOD). For example, septicaemia is often chosen as the underlying or precipitating COD when it is, in fact, the immediate cause arising from a many possible UCODs including communicable or non-communicable diseases, or an injury [3]. Prevention strategies would differ markedly depending on the UCOD; hence the importance of correct certification.

Garbage codes bias a country’s true pattern of mortality. Studies of the quality of mortality statistics carried out in Thailand [4], Sri Lanka [5], and Iran [6], for example, have repeatedly found that the population’s likely true mortality pattern was considerably different from the pattern reported by the CRVS system. These discrepancies have been largely attributed to physicians’ extensive use of garbage codes.

## Towards a more useful public health classification of garbage codes

The rationale for identifying garbage codes is that, by doing so, certifying physicians and coders can be encouraged and trained to avoid unspecific ICD codes that are unlikely to be useful in guiding disease and injury control strategies. Specifically, national health planners must understand which misdiagnoses have the greatest impact on policy decions. Rather than classifying garbage codes according to the type of error (see Naghavi et al. [3]), an alternative classification is therefore needed, based on the severity of the impact that particular garbage codes might have in seriously misinforming public policy.

Accordingly, we have adapted the classification of garbage codes used by the Global Burden of Disease study to guide efforts to improve CRVS data quality. We focus more on the likely policy implications of various types of misdiagnosis of the true UCOD. The four distinct levels of garbage codes are defined as:

- **Level 1 (very high) – codes with serious policy implications.** These are causes (e.g. septicaemia) for which the true UCOD might belong to any one of three broad cause groups: communicable or non-communicable diseases, or injuries. We simply don’t know. Such errors potentially grossly misinform understanding of the extent of an epidemiological transition in a population.
- **Level 2 (high) – codes with substantial policy implications.** These are causes for which the true UCOD is likely to belong to one, or at most two, of the three broad groups, (e.g., ‘essential (primary) hypertension’). While not greatly altering the understanding of the broad composition of mortality in a population, these codes might considerably affect the comparative importance of leading causes within broad disease categories.
- **Level 3 (medium) – codes with important policy implications.** These are causes for which the true underlying UCOD is likely to be within the same ICD chapter. For instance, ‘unspecified cancer’ still identifies the death as being attributed to cancer, thus has some policy value, although greater type (site) specificity is required because different strategies are applied for different sites of cancer (e.g. breast versus lung).
- **Level 4 (low) – codes with limited policy implications.** These are diagnoses for which the true UCOD is likely to be confined to a single disease or injury category (e.g. unspecified stroke would still be assigned as a stroke death). The implications of unusable causes classified at this level will therefore, generally, be much less important for public policy.

To better focus data quality improvement efforts, this new classification only identifies the garbage codes that are truly unhelpful for policy and are used frequently by physicians to certify deaths; namely, levels 1–3. This excludes, for example, ‘unspecified pneumonia’, which although considered a garbage code in the Global Burden of Disease study, given its relevance for research and technology development [4], can be ignored in this public health oriented framework since we believe it provides sufficient information to guide public health interventions. Morever, any public health-orientated garbage code classification must be realistic about countries’ diagnostic capacity at different levels of development. For example, to reliably distinguish between haemorrhagic and ischaemic stroke, a computed tomography scan or magnetic resonance image is usually necessary – technologies that are not widely available in low- and middle-income countries.

## Conclusion

### Implications for mortality data systems

To help countries identify the pattern and extent of garbage coding in their COD data, this new typology of garbage codes has been included in the data quality assessment tool, Analysis of Causes of National Deaths for Action (ANACONDA) developed by the University of Melbourne’s Bloomberg Philanthropies Data for Health Initiative in partnership with the Swiss Tropical and Public Health Institute at the University of Basel [7].

This tool allows countries to identify not only the relative importance of different categories of garbage codes, but also the ICD codes that are most commonly misused within each of these three levels. Strategies to improve COD data quality in hospitals should address the most commonly used garbage codes from all categories. However, clearly, greater emphasis should be given to reducing the frequency of those codes, which have the greatest potential to seriously distort the evidence-base for public health policy designed to reduce premature mortality.

## Data Availability

Not applicable.

## Abbreviations

COD: Cause of death
CRVS: Civil registration and vital statistics
ICD: International Statistical Classification of Diseases and Related Health Problems
UCOD: Underlying cause of death
